# Folic Acid Rescues Valproic Acid-Induced Morphogenesis Inhibition in Neural Rosettes Derived From Human Pluripotent Stem Cells

**DOI:** 10.3389/fncel.2022.888152

**Published:** 2022-05-16

**Authors:** Xiao-zuo Zhang, Hai-qin Huo, Yu-qing Zhu, Hao-yang Feng, Jiao Jiao, Jian-xin Tan, Yan Wang, Ping Hu, Zheng-feng Xu

**Affiliations:** Department of Prenatal Diagnosis, The Affiliated Obstetrics and Gynecology Hospital of Nanjing Medical University, Nanjing Maternity and Child Health Care Hospital, Nanjing, China

**Keywords:** neural tube defects, human pluripotent stem cells, neural rosette, valproic acid, folic acid

## Abstract

The ability of human pluripotent stem cells (hPSCs) to specialize in neuroepithelial tissue makes them ideal candidates for use in the disease models of neural tube defects. In this study, we cultured hPSCs in suspension with modified neural induction method, and immunostaining was applied to detect important markers associated with cell fate and morphogenesis to verify the establishment of the neural tube model *in vitro*. We carried out the drug experiments to further investigate the toxicity of valproic acid (VPA) exposure and the potential protective effect of folic acid (FA). The results demonstrated that neural rosette undergoes cell fate speciation and lumen formation accompanied by a spatiotemporal shift in the expression patterns of cadherin, indicating the model was successfully established. The results showed that VPA caused morphogenesis inhibition of lumen formation by altering cytoskeletal function and cell polarization, which could be rescued by FA supplement.

## Introduction

Neural tube defects (NTDs) are one of the most common congenital malformations. The main manifestations include spina bifida and anencephaly (Avagliano et al., 2019). Severe fatal NTDs lead to death during the fetal period, while surviving children have to face lifelong disability as well as the high cost of medical and life care, which brings the family and society heavy burden (Malcoe et al., 1999). NTDs are caused by the interaction of environmental and genetic factors. The environmental factors include medicine exposure, infection, and radiation (Jia et al., 2019). Valproic acid (VPA) is one of the most commonly prescribed drugs to control epileptic seizures. Unfortunately, reports have shown that VPA exposure during pregnancy increases the risk of NTDs in the fetus, which puts the use of VPA in pregnant women into a dilemma (Diav-Citrin et al., 2008; Ornoy, 2009). Therefore, exploring the mechanism of NTDs induced by VPA is of great significance.

The current research on NTDs is mainly based on animal models, which has confirmed that VPA is neurotoxic to embryonic development (Hughes et al., 2018). Neurodevelopmental toxicity of VPA in zebrafish embryos includes developmental delay (Teixidó et al., 2013), brain defects (Cowden et al., 2012), locomotor behavioral changes, and social interaction impairment (Dwivedi et al., 2019). Although a detailed neurotoxic analysis of VPA exposure has been performed, the mechanism by which VPA is teratogenic remains unknown, and a range of potential mechanisms including folic acid (FA) antagonism have been proposed (Dawson et al., 2006; Fathe et al., 2014; Semmler et al., 2017).

Folic acid is an essential synthetic nutritional supplement found in fortified food and dietary supplements. FA metabolism is vital for nervous system development and function, enabling nucleic acid biosynthesis, cell proliferation, and growth necessary for the neurogenesis process (Balashova et al., 2018). With the promotion of FA supplementation during pregnancy, the prevalence of NTDs has reduced significantly (Czeizel et al., 2013), which reduces the incidence of NTDs in humans by 60–70% (van der Linden et al., 2006)and rescues NTDs induced in chick embryos (Güney et al., 2003; Weil et al., 2004), whereas the specific protective mechanism is not yet clear. FA pathway is of considerable importance, as clinicians need to know if FA supplementation can prevent VPA-induced NTDs. It is well established that FA supplementation reduces the risk of embryonic NTDs and other congenital malformations in humans and rodents (Dawson et al., 2006). As VPA-induced neurotoxicity is assumed to be related to folate deficiency (Semmler et al., 2017), we hypothesized that FA is a feasible candidate for preventing VPA-induced embryonic neurogenesis abnormalities.

Model animals such as mice, chickens, and zebrafish have certain limitations in exploring the mechanism of human NTDs induced by VPA for substantial interspecies divergence. Human pluripotent stem cells (hPSCs) are widely employed in the study of human embryonic development for their self-renewal and multi-differentiation potential characteristics (Takahashi et al., 2007), which offer a unique window to explore the process of early embryonic development. In this study, we modified neural induction method to generate neural rosette tissue with a single lumen derived from hPSCs (Meinhardt et al., 2014), morphologically similar to the pseudo-stratified columnar epithelium of neural tube *in vivo*, which can be used as a stable neural tube model *in vitro*. Based on the model, we explored the effect of VPA exposure on neural differentiation to further probe the potential mechanism underlying VPA-induced NTDs. Finally, we investigated whether FA could rescue VPA-induced perturbations.

## Materials and Methods

### Human Pluripotent Stem Cells Culture and Neural Induction

Undifferentiated hPSCs (WA-09 alias H9, WiCell) were seeded into Matrigel-coated 6 well plates in media (Thermo Fisher, CA1001500) without feeders and passaged weekly. HPSCs were differentiated into neural rosettes using a published method with minor modifications (Meinhardt et al., 2014)and cultured in neural induction media (NIM) containing N2(1:100), NEAA(1:100), and DMEM/F12. Y-27632 (10 μM) and B27(1:50) supplement were added into the NIM at day 0 of differentiation. Fresh NIM medium was freshly exchanged daily.

### Immunofluorescence Staining

Tissues were fixed in 4% paraformaldehyde at 4°C for 5 h before dehydration and then embedded in O.C.T for the frozen slice. Tissues were permeabilized with 0.2% Triton X100 in PBS for 10 min followed by blocking with 10% donkey serum in PBS for 1 h at room temperature. Primary antibodies were applied overnight at 4°C, followed by incubation with corresponding secondary antibodies-conjugated with Alexa Fluor 488 or 546 at room temperature for 1 h on the next day. The primary antibodies we used in the study are NANOG (R&D, AF1997), PAX6 (Biolegend, 901302), ECAD (Cell Signaling, 3195T), NCAD (Biolegend, 350802), SOX2 (R&D, AF12018), OCT4 (Abcam, ab181557), Nestin (Santa Cruz, sc-23927), PHH3 (Millipore, MABE941), ZO-1 (Thermo Fisher, 61-7300), PKCλ (BD, 610207), EZRIN (Sigma, E8897), PAX3(R&D, MAB2457), and NKX2.1(Santa Cruz, sc-13040). The stained coverslips were mounted for confocal laser scanning microscopy. We randomly select different positions for fluorescence intensity measurement with ZEN and performed intensity analysis with GraphPad Prism 7.

### Western Blot

Proteins were extracted from tissue with RIPA buffer (Sigma, R0278) containing protease inhibitor (Roche, 04693159001). The concentrations of extracted cellular proteins were measured using a QuantiPro BCA assay kit (Sigma, QPBCA). Equal amounts of protein were loaded into SDS-PAGE gels and transferred to a nitrocellulose membrane with Transfer-Blot Turbo system (Bio-rad, 170-4150). The membranes were blocked with 5% skim milk in TBST and the primary antibody phosphorylated myosin light chain (pMLC) (Cell Signaling, 3671) was incubated overnight at 4°C. After incubation with secondary antibodies conjugated with horseradish peroxidase, protein bands were visualized using the G: Box Bio Imaging systems. Protein expression was normalized to GAPDH (Abcam, ab181602), which serves as a loading control.

### RNA Sequence

The purity of the sample was determined by NanoPhotometer (IMPLEN, CA, United States). The concentration and integrity of RNA samples were detected by Agilent 2100 RNA Nano 6000 Assay Kit (Agilent Technologies, CA, United States). Sequencing libraries were generated using VAHTS Universal V6 RNA-seq Library Prep Kit for Illumina (NR604-01/02). RNA concentration of library was measured using Qubit RNA Assay Kit in Qubit 3.0 to preliminary quantify and then dilute to 1 ng/μl. Insert size was assessed using the Agilent Bioanalyzer 2100 system (Agilent Technologies, CA, United States). After the insert size met the expectation, the Bio-RAD CFX 96 fluorescence quantitative PCR instrument was used to accurately quantify the library effective concentration, and the reagent used was Bio-RAD KIT iQ SYBR GRN. The clustering of the index-coded samples was performed on a cBot cluster generation system using HiSeq PE Cluster Kit v4-cBot-HS (Illumina) according to the manufacturer’s instructions. After cluster generation, the libraries were sequenced on an Illumina platform and 150 bp paired-end reads were generated. The cluster generation and sequencing were performed on Novaseq 6000 S4 platform, using NovaSeq 6000 S4 Reagent kit V1.5 (Annoroad Gene Technology, Beijing, China). Each group of samples includes control group and VPA1 μM group and the experiment was performed in three biological replicates.

### Statistical Analysis

GraphPad Prism 7 (GraphPad Software) was used for all statistical analyses and graphs made. Statistical analysis was performed by one-way ANOVA with *P*-values < 0.05 deemed significant. ns – *P* > 0.05, * – *P* < 0.05.

## Results

### Generation of the Neural Rosette *in vitro*

**FIGURE 1:**
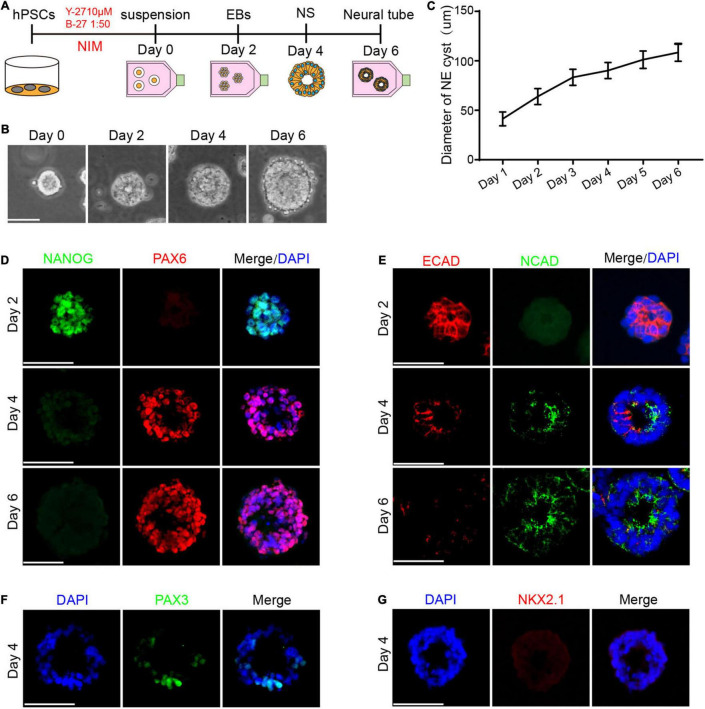
Cell fate specialization of the neural rosette. **(A)** A schematic diagram illustrating the method of differentiating hPSCs to the neural rosette (Meinhardt et al., 2014). **(B)** Morphological results showed that the neural rosette contains a central cavity. **(C)** The diameter of cell aggregates from day 1 to day 6, *n* = 25. **(D)** The expression of NANOG decreased and the expression of PAX6 increased during differentiation. **(E)** There was a transition of the temporal-spatial expression pattern of ECAD and NCAD during the differentiation. **(F)** The expression of PAX3 at day 4. **(G)** The expression of NKX2.1 at day 4. Scale bars: 50 μm.

We first modified the neural induction method to obtain a single neural rosette derived from hPSCs (Figure 1A). Initially, hPSCs were dissociated into a single cell and cultured in suspension in the NIM containing N2(1:100), NEAA(1:100), and DMEM/F12, which generated free-floating cell aggregates by day 2, defined as embryoid bodies (EBs). We noted the appearance of the neural rosette with a central cavity by day 4, which further differentiated into radial tissue composed of multi-layered cells by day 6 (Figure 1B). As observed, the morphological analysis revealed that the diameter of cell aggregates gradually increased during the differentiation process (Figure 1C).

### Cell Fate Specialization During the Differentiation

The development of early embryo involves a pluripotent cell population within the inner cell mass differentiating to the epiblast and then to the three germ layers, ectoderm, mesoderm, and endoderm. The embryonic ectoderm thickens to form pseudostratified columnar cells and extends to form a neural plate under neural induction, which folds to the dorsal of the embryo to form the neural tube (Chizhikov and Millen, 2005). To verify the characteristic of the neural rosette, we first examined the tissue from the aspect of cell fate specialization.

NANOG is known as a master regulator of self-renewal and pluripotency, and the downregulated level of which leads to the exit from pluripotency and ultimately differentiation (Pan and Thomson, 2007). According to the development process *in vivo*, we first detected the expression of NANOG. Immunofluorescence (IF) staining showed that almost all cells in the EBs expressed NANOG by day 2. As expected, we observed a strong reduction in the level of NANOG from day 2 to day 6 (Figure 1D), which demonstrated that hPSCs have gradually exited from pluripotency under neural induction. Ectodermal lineage marker PAX6 plays an important role in regulating neurogenesis during the development of neural tube (Osumi et al., 2008). Therefore, we examined the expression of PAX6 during the differentiation. IF staining revealed that there was no expression of PAX6 by day 2, while PAX6 was detected in the neural rosette by day 4 and most of the cells were PAX6 positive by day 6 (Figure 1D), indicating that neural rosette has differentiated into neuroectoderm.

After specification of the neuroectoderm at the dorsal ectoderm germ layer, the development of neural tube continues with the acquisition of dorsal neural identity, revealed by expression of dorsal markers such as PAX3, by the midline dorsal ectodermal cells of the gastrulating embryo. We thus sought to examine the dorsal and ventral fate of neural rosette derived from hPSCs. Immunostaining revealed that neural rosette expressed the dorsal neural marker PAX3 at day 4 (Figure 1F). PAX6 are expressed by undifferentiated cells in the ventral region of the neural tube (Ericson et al., 1997). We detected the expression of PAX6 at day 4 while the ventral neural marker NKX2.1 was not detectable at day 4 (Figure 1G). In the previous research, PAX6 is first detected at 5 somites (Inoue et al., 2000; Bell et al., 2001) while NKX2.1 expression is initially observed slightly later at 10–12 somites (Shimamura et al., 1995; Sussel et al., 1999). Together, these data showed the dorsal-ventral pattern of neural rosette derived from hPSCs is consistent with the previous research.

**FIGURE 2:**
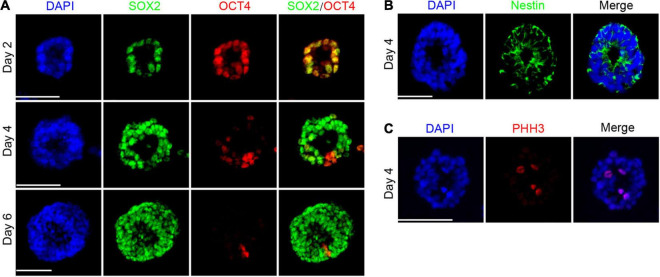
The appearance of pseudo-stratified columnar epithelium during cell fate specialization. **(A)** The separation of SOX2 and OCT4 during the differentiation. **(B)** Neural rosette expressed Nestin by day 4. **(C)** Expression of PHH3 was rich at the apical area by day 4. Scale bars: 50 μm.

To further understand the process of cell fate specialization, we examined crucial markers including early neuroectoderm marker SOX2 (Kishi et al., 2000) and pluripotency marker OCT4 highly expressed in the inner cell mass and epiblast of the embryo (Pesce and Schöler, 2001). As expected, most cells expressed SOX2 and OCT4 simultaneously by day 2, consistent with the developmental phenomenon that SOX2 expression is earlier than PAX6 in the gastrulation stage *in vivo*. We found the separation of SOX2 and OCT4 in the neural rosette and there were two types of cells: SOX2 + OCT4- and SOX2-OCT4+. The proportion of SOX2-OCT4 + cells accounted for only a small part (Figure 2A), indicating that the loss of pluripotency and acquisition of neural property drive hPSCs toward neuroectoderm. In addition, Nestin expressed by neural stem cells was positive in the neural rosette and formed pseudo-stratified columnar epithelium (Figure 2B), similar to previous research. Phospho-Histone H3 (PHH3), a recently described marker specific for proliferation (Bershteyn et al., 2017), was preferentially distributed at the apical domain of single-layered neuroepithelium (Figure 2C).

### Temporal-Spatial Transformation of Cadherins

The previous study has revealed that the transformation of cadherin during embryonic development plays a crucial role in regulating cell fate decisions. We detected the expression of E-cadherin (ECAD) and N-cadherin (NCAD), typical Type I cadherins with temporal-spatial expression patterns. IF staining showed that ECAD was highly expressed in the intercellular space by day 2, and there was faint expression by day 4 (Figure 1E), similar to that of high expression on the cell surface during the HH8 period in the chicken embryo (Rogers et al., 2018). Meanwhile, no expression of NCAD was detected by day 2 and we found that NCAD was enriched at the apical part in the neural rosette tissue by day 4, which formed a closed ring in the center (Figure 1E), consistent with the phenomenon that NCAD was highly expressed in the neural tube during the HH10 period in the chicken embryo (Rogers et al., 2018). HH8 and HH10 were a series of normal stage in the development of the chick embryo proposed by Hamburger and Hamilton (1992).

### The Formation of a Single Lumen

**FIGURE 3:**
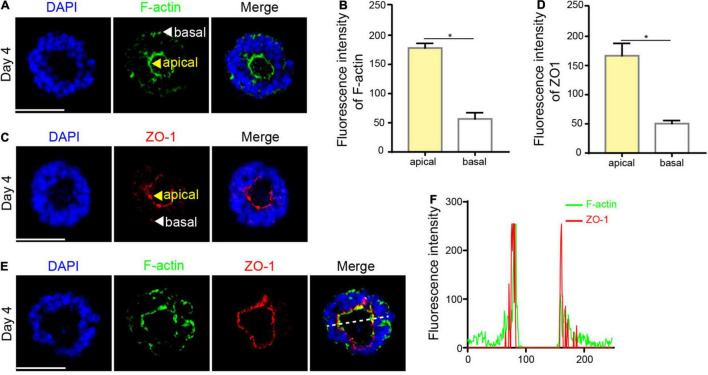
The formation of a single lumen. **(A,B)** F-actin was concentrated at the apical area in the neural rosette. **(C,D)** ZO-1 was concentrated at the apical area in the neural rosette. **(E)** F-actin co-localized with ZO-1 at the apical area. **(F)** The fluorescence intensity of F-actin and ZO-1. Scale bars: 50 μm. *P < 0.05.

The apical ring of the neural rosette is highly similar to the lumen of the neural tube *in vivo*, which is a crucial morphological event during neural tube development. The myosin at the apical domain of neuroepithelium assembles and contracts, defined as apical constriction (Morita et al., 2010). Therefore, we examined the expression of cytoskeleton marker F-actin. IF staining showed that F-actin was concentrated at the apical area in the neural rosette t (Figures 3A,B). Apart from contractile actomyosin networks at the apical domains, lumen morphogenesis requires intercellular tight junctions (Nishimura et al., 2012). ZO-1 is a membrane peripheral protein related to the tight junctions between cells. Thus, we next examined the expression of ZO-1. As expected, ZO-1 was enriched at the apical surface in the neural rosette (Figures 3C,D), similar to the distribution of F-actin. Besides, the fluorescence intensity of F-actin and ZO-1 presented a coincident double-peak shape, in which a low signal area represents lumen, indicating that F-actin co-localizes with ZO-1 at the apical area (Figures 3E,F), consistent with the previous study (Odenwald et al., 2017).

**FIGURE 4:**
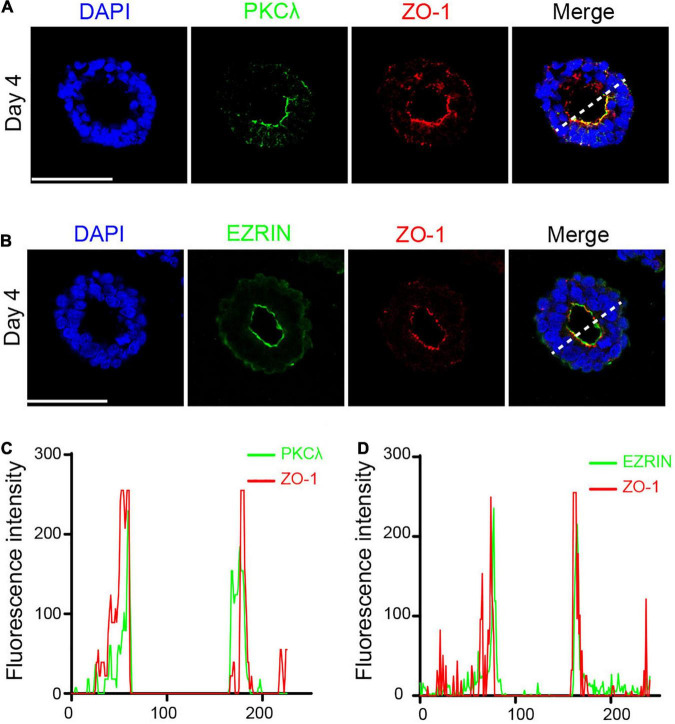
Polarized protein co-localized with ZO-1 at the apical area. **(A)** PKCλ was enriched at the apical part of epithelial cells. **(B)** EZRIN was enriched at the apical part of epithelial cells. **(C,D)** PKCλ and EZRIN co-localized with ZO-1 at the apical area. Scale bars: 50 μm.

Simultaneously, the polarized protein that interacts with the cytoskeleton is enriched at the luminal surface, leading to asymmetric division of neuroepithelial cells. PKCλ is a core protein that maintains the polarity and integrity of neuroepithelial cells (Chen et al., 2017). EZRIN interacts with both F-actin and membrane proteins, exhibiting strong polarity of the actin cytoskeleton (Zhu et al., 2020). To further examine the emergence of polarized neuroepithelial tissues, we continued to detect the expression of PKCλ and EZRIN in the neural rosette. IF staining showed that PKCλ was enriched at the apical part of epithelial cells, generating a polarized ring (Figure 4A), consistent with what was previously reported (Chen et al., 2017). Meanwhile, we found the existence of the EZRIN-rich apical domain in the neural rosette (Figure 4B). In addition, fluorescence intensity analysis revealed that both PKCλ and EZRIN co-localized with ZO-1 at the apical area, presenting a double-peak shape (Figures 4C,D).

All above results demonstrated that the neural rosette derived from hPSCs we have obtained could be used as a neural tube model *in vitro*, providing a research platform to explore the mechanism of NTDs.

### Valproic Acid-Induced Morphogenesis Inhibition on Neural Tube Model

To explore the effect of VPA exposure on neural tube development, hPSCs were treated with increasing concentrations of VPA at 0 μM (control), 0.1 μM, 1 μM, and 10 μM from day 0 (Supplementary Figure 1A). Compared to control, the neural tube in the VPA 10 μM group was irregular with messy structure; the neural tube in the VPA 1 μM group was visible with ambiguous structure. There was no significant difference between the VPA 0.1 μM group and the control group. The diameter displayed a decrease in a dose-dependent manner (Supplementary Figures 1B,C). These results implied that VPA induced morphogenesis inhibition on the neural tube model.

**FIGURE 5:**
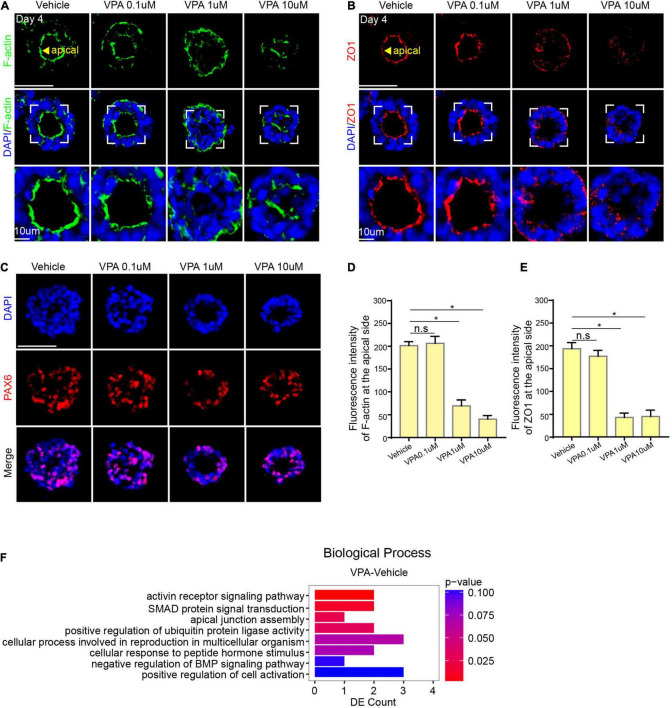
VPA-induced morphogenesis inhibition on neural tube model. **(A,D)** The expression of F-actin at the apical domain in control, 0.1, 1, and 10 μM group. **(C)** The expression of PAX6 in control, 0.1, 1, and 10 μM group. **(B,E)** The expression of ZO-1 at the apical domain in control, 0.1, 1, and 10 μM group. **(F)** RNA sequencing of VPA1 μM and control group. Scale bars: 50 μm. *P < 0.05.

Since VPA was added on day 0 of the differentiation process, it is particularly necessary to determine that whether VPA blocked the induction of neuroepithelial lineages. We have already characterized the neurogenesis in the model under control conditions, thus we examined the expression of PAX6 in the VPA model. Immunostaining showed that the expression of PAX6 was positive in both control group and VPA-treated groups (Figure 5C), representing a success of EBs to differentiate into neuroepithelial lineages.

Structure damage is related to lumen formation, a crucial morphological event during the differentiation *in vitro*. To further investigate how VPA impedes lumen morphogenesis, we first detected the expression of F-actin. IF staining showed that the expression of F-actin in VPA-treated groups except the VPA 0.1 μM group was in a disorderly distribution and failed to accumulate at the apical surface (Figures 5A,D). We next detected ZO-1 and found that there was a faint expression of ZO-1 at the apical surface in VPA 10 μM and VPA 1 μM groups (Figures 5B,E). These results demonstrated that VPA has a destructive effect on the cytoskeleton reorganization and cell polarization, which may be the mechanism of VPA-induced morphogenesis inhibition.

The phenotyping results above showed that VPA can impede lumen formation of the neural rosette. RNA sequencing (RNA-seq) was performed to gain insight into the underlying molecular basis for the changes. Gene Ontology (GO) analysis suggested genes differentially expressed were enriched in several biological processes including apical junction assembly (Figure 5F). These gene expression changes indicated that VPA exposure might affect apical constriction.

### Folic Acid-Mediated Remedy for Valproic Acid-Induced Morphogenesis Inhibition

Studies have found that FA has a protective effect on the neurotoxicity of VPA (Muhsen et al., 2021), we hypothesized that FA is a feasible candidate for preventing VPA-induced embryonic neurogenesis abnormalities. To further investigate whether FA supplementation could ameliorate the VPA-induced negative effect, we cotreated hPSCs with VPA1 μM and FA10 μM, defined as FV group (Supplementary Figure 2A). It is noteworthy that there was a visible structure in the FV group and there was no significant difference between the control and FV group (*P* > 0.05) (Supplementary Figures 2B,C), indicating that FA rescued VPA-induced morphogenesis inhibition.

**FIGURE 6:**
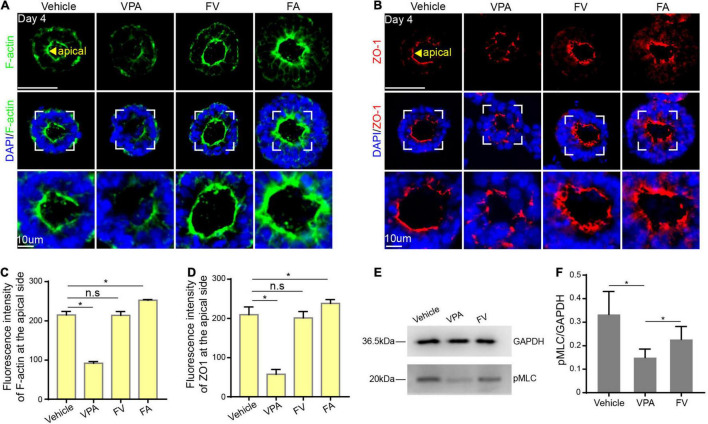
FA rescued VPA-induced morphogenesis inhibition. **(A,C)** The expression of F-actin at the apical domain in control, VPA1 μM, FV and FA10 μM group. **(B,D)** The expression of ZO-1 at the apical domain in control, VPA1 μM, FV, and FA10 μM group. **(E)** Western blot of pMLC in control, VPA1 μM, FV group. **(F)** Quantification of triplicate experiments. Scale bars: 50 μm. *P < 0.05.

To further explore whether FA protects against VPA-induced destruction of cytoskeleton reorganization and cell polarization, we first detected the expression of F-actin and found that the expression of F-actin in the FV group was condensed at the apical surface compared to the VPA1 μM group (Figures 6A,C). In addition, we examined the expression of ZO-1. There was a similar expression pattern of ZO-1 between the FV and control group (Figures 6B,D). The above results indicated that FA counteracts VPA-induced damage on cytoskeleton reorganization and cell polarization.

To investigate the molecular mechanism underlying the protective effect, western blot was applied to detect the expression of pMLC, an active form of myosin II, closely related to apical constriction. Results demonstrated that the expression of pMLC in the VPA 1 μM group was decreased compared to the control. When co-treated with VPA and FA, the expression of pMLC increased (Figures 6E,F), suggesting that FA rescued the VPA-induced morphogenesis inhibition by increasing the level of pMLC expression.

## Discussion

In the present study, we first modified the neural induction method to generate neural rosette and found that there were cell fate specialization and lumen formation accompanied with the temporal-spatial expression pattern of cadherins. Therefore, we hypothesized that neural rosette derived from hPSCs can be used as a neural tube model *in vitro* to assess VPA neural teratogenicity, which is rapid and effective and avoids ethical restriction and interspecies divergence compared to animal models.

Here, we demonstrated that VPA exposure resulted in a dose-dependent morphogenesis inhibition on the neural tube model. Disordered distribution of F-actin and ZO-1 revealed that VPA exposure destroyed lumen formation by impairing cytoskeleton reorganization and cell polarization. RNA-seq indicated that VPA exposure affected the expression of genes closely related to apical contraction, which is required for lumen formation in neural tube morphogenesis, a hallmark of vertebrate epithelial cell layers. Apical constriction of cells depends upon the actin-myosin contractile force driven by localized myosin light chain phosphorylation. Actin polymerization plays an important role in apical contraction which promotes cytoskeletal organization (Taniguchi et al., 2015). Claudins are essential for cell shape changes during neural tube closure. Their removal did not affect cell type differentiation, neural ectoderm patterning (Baumholtz et al., 2017). Further investigations are required to make a distinction between these mechanisms.

It is worth noting that we observed that FA supplementation counteracts the VPA-induced effect on the cytoskeleton and cell polarity by upregulating the expression of pMLC. In a previous study, VPA-induced neural teratogenicity is linked to perturbations in neurogenesis-related FA pathway in folate-deficient models (Kao et al., 2014) and FA protects against VPA-induced neural tube and brain defects in mice (Dawson et al., 2006) and rats (Shona et al., 2018). Therefore, our results are consistent with the above studies. Interestingly, pMLC is an activated form of myosin II phosphorylated by MLCK (Ikebe and Hartshorne, 1985). A recent study suggests that FA improves the shape of epithelial cells during morphogenesis through the MLCK pathway (Martin et al., 2019), which is a possible mechanism for FA to play a rescue role. On the other hand, there are conflicting findings suggesting that FA supplementation has no protective effects against VPA-induced neural teratogenicity in mice (Hansen et al., 1995) and case-control studies in humans (Jentink et al., 2010). Further research is required to decipher the mechanism underlying the protective effect. In addition, the molecular interactions that regulate many of the morphogenetic changes required for neural tube closure occur at the apical cell surface (Colas and Schoenwolf, 2001; Lawson et al., 2001). During neural tube closure, apical localization of RhoA/ROCK signaling components at the neural plate midline is required for pMLC, the downstream target of RhoA/ROCK signaling, which then moves along actin filaments to generate the contractile force required for apical constriction (van Straaten et al., 2002). We speculate that FA may affect the expression of pMLC by RhoA/ROCK signaling. Further research is required to decipher the mechanism underlying the protective effect.

To conclude, we established an effective neural tube model *in vitro* from hPSCs by modifying the published method. Based on the model, we found that VPA induced morphogenesis inhibition including the destruction of cytoskeleton function and cell polarity, and FA supplementation rescued these perturbations. Combining our results and existing research, we assume that the mechanism of FA-mediated remedy for VPA-induced perturbations may be associated with the MLCK pathway, further molecular mechanism remains uncertain. Furthermore, we are not sure whether there is a decrease in the percentage of MLC phosphorylation or the total levels of MLC by quantifying pMLC with GAPDH as a control. It is also unclear whether pMLC is a cause or consequence of the observed failure of lumen formation, thus more research needs to be performed. In addition, excessive apoptosis is an underlying cause of NTDs (Huang et al., 2019). Apoptosis is known to occur during and after neurulation in the neuroepithelium, although the significance of this finding is not fully understood. It has been postulated that apoptosis is required for bending of the neural folds at the dorsolateral hinge point and for midline epithelial remodeling once the neural folds have come into contact and fused (Copp et al., 2003). Another study has shown that apoptosis is not a requirement for neural tube closure (Massa et al., 2009). Nevertheless, excessive cell death in the neuroepithelium can disrupt anterior neural tube closure by leaving the embryo with an inadequate number of cells to undergo proper closure. Absence of exploration of apoptosis in the neuroepithelium caused by VPA was another limitation in the study.

## Data Availability Statement

The data presented in the study are deposited in the NCBI SRA database repository, accession number: PRJNA824135.

## Author Contributions

X-ZZ and H-QH designed the study. X-ZZ performed the experiments and wrote the draft manuscript. Y-QZ, JJ, and H-YF participated in the manuscript modification. J-XT and YW performed the data analysis. Z-FX and PH revised the article. All authors accepted the final manuscript.

## Conflict of Interest

The authors declare that the research was conducted in the absence of any commercial or financial relationships that could be construed as a potential conflict of interest.

## Publisher’s Note

All claims expressed in this article are solely those of the authors and do not necessarily represent those of their affiliated organizations, or those of the publisher, the editors and the reviewers. Any product that may be evaluated in this article, or claim that may be made by its manufacturer, is not guaranteed or endorsed by the publisher.
