# Supplementary material for: Folic Acid Rescues Valproic Acid-Induced Morphogenesis Inhibition in Neural Rosettes Derived From Human Pluripotent Stem Cells
Source: Front Cell Neurosci. 2022 May 16;16:888152. doi: 10.3389/fncel.2022.888152 (PMC9148965; doi:10.3389/fncel.2022.888152)
Supplement: Supplementary file 1 [file Data_Sheet_1.docx]

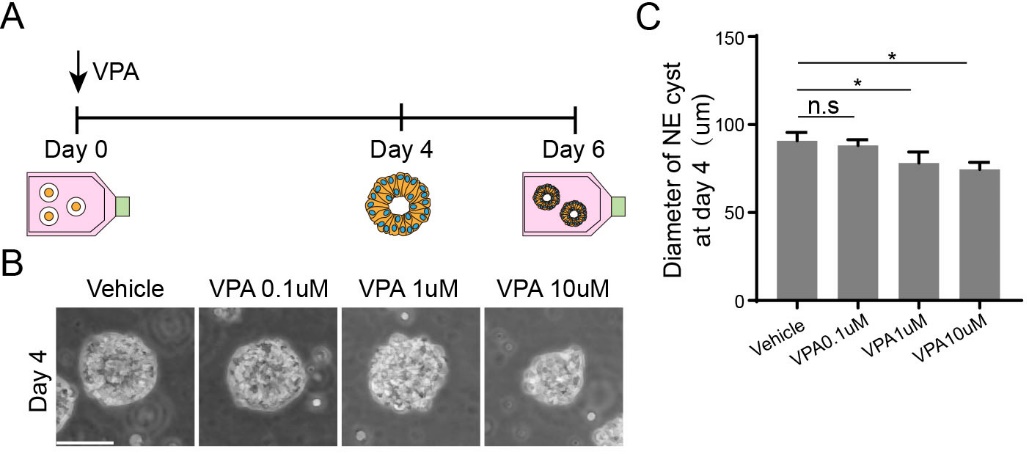


**FIGURE S1** VPA-induced inhibition on neural tube model. **(A)** hPSCs were treated with VPA from day 0. **(B)** Morphological results of control, 0.1μM, 1μM, and 10μM group. **(C)** The diameter of the neural rosette in control, 0.1μM, 1μM, and 10μM group. Scale bars:50μm. **P*<0.05.


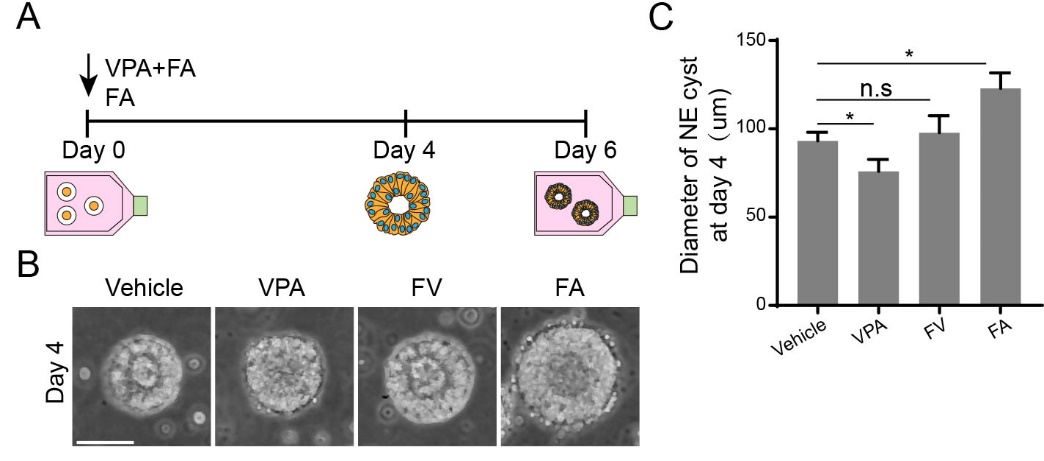


**FIGURE S2** FA rescued VPA-induced inhibition on neural tube model. **(A)** hPSCs were co-treated with VPA and FA from day 0. **(B)** Morphological results of control, VPA1μM, FV group, and FA10μM group. **(C)** The diameter of the neural rosette in control, VPA1μM, FV group, and FA10μM group. Scale bars:50μm. **P*<0.05.
